# Pitfalls and Rewards of Setting Up a Liquid Biopsy Approach for the Detection of Driver Mutations in Circulating Tumor DNAs: Our Institutional Experience

**DOI:** 10.3390/jpm12111845

**Published:** 2022-11-04

**Authors:** Michelle Chen, Damon Jian, Maxim Sidorov, Rinette W. L. Woo, Angela Kim, David E. Stone, Ari Nazarian, Mehdi Nosrati, Ryan J. Ice, David de Semir, Altaf A. Dar, Roman Luštrik, Janez Kokošar, Luka Ausec, Michael C. Rowbotham, Gregory J. Tranah, Mohammed Kashani-Sabet, Liliana Soroceanu, Sean D. McAllister, Pierre-Yves Desprez

**Affiliations:** 1 California Pacific Medical Center, Research Institute, 475 Brannan Street, Suite 130, San Francisco, CA 94107, USA; 2 Genialis Inc., 177 Huntington Ave, Ste 1703, Boston, MA 02115, USA

**Keywords:** cancer, metastasis, plasma, next-generation sequencing

## Abstract

We describe our institutional experience of developing a liquid biopsy approach using circulating tumor DNA (ctDNA) analysis for personalized medicine in cancer patients, focusing on the hurdles encountered during the multistep process in order to benefit other investigators wishing to set up this type of study in their institution. Blood samples were collected at the time of cancer surgery from 209 patients with one of nine different cancer types. Extracted tumor DNA and circulating cell-free DNA were sequenced using cancer-specific panels and the Illumina MiSeq machine. Almost half of the pairs investigated were uninformative, mostly because there was no trackable pathogenic mutation detected in the original tumor. The pairs with interpretable data corresponded to 107 patients. Analysis of 48 gene sequences common to both panels was performed and revealed that about 40% of these pairs contained at least one driver mutation detected in the DNA extracted from plasma. Here, we describe the choice of our overall approach, the selection of the cancer panels, and the difficulties encountered during the multistep process, including the use of several tumor types and in the data analysis. We also describe some case reports using longitudinal samples, illustrating the potential advantages and rewards in performing ctDNA sequencing to monitor tumor burden or guide treatment for cancer patients.

## 1. Introduction

Compared with classic tumor biopsies, liquid biopsies are more convenient, easily obtainable, and present minimal procedure risks to patients. Circulating tumor DNA (ctDNA) in peripheral blood contains gene mutations found in primary tumors, and serial sampling of ctDNA can have diagnostic value, and predict response to treatment and clinical outcome. Previous studies have shown the potential power of this approach to monitor tumor burden in cancer patients [1,2,3]. Tumor types such as melanoma are particularly amenable to ctDNA analysis given the high mutational burden observed [4].

To date, the utility of extracting and sequencing ctDNA (a subpopulation of circulating cell-free DNA (ccfDNA) corresponding to DNA shed by cancer cells) has been demonstrated in a variety of tumor types. For example, next-generation sequencing (NGS) data were collected from 145 patients with about 25 different types of cancer, including the two major subtypes of lung cancer, and *TP53* alterations comprised a significant proportion of the mutation pool [5]. Moreover, the pathological variants in ctDNA samples from 60 colorectal patients were studied by NGS. The authors concluded that ctDNA analytical pipeline had satisfactory sensitivity and specificity that could be used in the postoperative surveillance of colorectal cancer [6].

In a study involving breast cancer patients with both tumor and plasma sequencing data, 14 patients had concordant/partially concordant mutations in plasma and tumor, whereas 16 patients had mutations only in tumors with negative ctDNA [7]. Using a large NGS panel that covers 1021 cancer-related genes, it was found that ctDNA could be used to predict tumor response to neoadjuvant therapy. Moreover, actionable mutations were identified in the ctDNA of 41 of 100 patients who had various types of tumors (around 12 different disease types) [8].

Besides NGS, digital-droplet PCR was used as another approach to detect the pancreatic ductal adenocarcinoma-associated somatic *KRAS* mutations in liquid biopsies from 59 patients. ctDNA was detected preoperatively in 49% of patients and was an independent predictor of overall survival. The authors concluded that measurement of *KRAS* ctDNA could be used to predict disease recurrence in pancreatic cancer patients [9].

Baseline and postoperative ctDNA detection identified stage III melanoma patients at highest risk of relapse and had the potential to inform adjuvant therapy decisions. In addition, a multicenter phase II trial of dabrafenib in *BRAF*(V600E/K) mutation-positive metastatic melanoma revealed that baseline ctDNA levels correlated with response rate and progression-free survival [10]. In another study, a high degree of concordance in *BRAF* mutation was observed between plasma and tissue [11]. Collectively, these results suggest the potential of ctDNA analysis in the monitoring of disease progression and treatment response in individual patients.

Despite these encouraging results, to date, few studies have examined the utility of a given ctDNA platform in both tissue and blood across multiple distinct solid tumors. The overall aim of our study is to describe the approaches chosen to reach the ultimate goal of tracking tumor progression and treatment response. As a first step towards this goal, we report here the results of a study initiated at our medical center comparing the mutations in tumor DNA and in DNA extracted from plasma obtained from patients with nine different cancer types. Blood samples were collected at time of tumor collection from patients with melanoma, pancreatic cancer, colorectal cancer, glioblastoma (GBM), ovarian cancer, lung cancer, cholangiocarcinoma, liver cancer, and breast cancer.

We describe the choice of the approach in our institution, the selection of the cancer panels, the hurdles encountered during the multistep process, including the use of several tumor types and data analysis. We also briefly describe the advantages of using these approaches during the collection of longitudinal samples obtained from the same patient.

## 2. Materials and Methods

### 2.1. Tumor DNA Extraction

DNA was extracted from 209 fresh frozen tissues obtained from patients with nine different types of cancer. Informed consent was obtained from all subjects involved in the study, which was conducted according to the guidelines of the Declaration of Helsinki, and approved by the Institutional Review Board of Sutter Health (protocol code 2015.059-1 approved on 3 October 2022). Isolation of genomic DNA was performed using the DNeasy tissue kit (Qiagen, Redwood City, CA, USA) after tissue disruption using ruptor disposable probes, and DNA was quantified using PicoGreen (Thermo Fisher Scientific, South San Francisco, CA, USA). DNA integrity was determined using agarose gels.

### 2.2. Circulating Cell-Free DNA Extraction

Blood samples were collected in tubes (PAXGene Blood Tubes that prevent hemolysis) with preservatives to increase shelf life. Extraction was performed on 209 human plasma samples (2–4 mL for each sample) stored at −80 °C. ccfDNA (which correspond to DNA fragments shed by all cell types including cancer cells) was isolated using QIAamp Circulating Nucleic Acid kit (Qiagen, Redwood City, CA, USA), and quantified using PicoGreen (Thermo Fisher Scientific, South San Francisco, CA, USA). After extraction, the samples were stored at −20 °C.

### 2.3. Next Generation Sequencing

Originally, DNA extracted from tumor samples was analyzed using the Illumina Truseq Amplicon Cancer panel (Foster City, CA, USA). MiSeq 2x151 base paired-end sequencing was performed to detect single-nucleotide variant (SNV) and insertion/deletion (indel) variants at 5% allelic frequency or higher in target regions with sufficient read coverage. The gene targets covered by the TruSeq Amplicon Cancer panel were as follows: *ABL1*, *AKT1*, *ALK*, *APC*, *ATM*, *BRAF*, *CDH1*, *CDKN2A*, *CSF1R*, *CTNNB1*, *EGFR*, *ERBB2*, *ERBB4*, *FBXW7*, *FGFR1*, *FGFR2*, *FGFR3*, *FLT3*, *GNA11*, *GNAQ*, *GNAS*, *HNF1A*, *HRAS*, *IDH1*, *JAK2*, *JAK3*, *KDR*, *KIT*, *KRAS*, *MET*, *MLH1*, *MPL*, *NOTCH1*, *NPM1*, *NRAS*, *PDGFRA*, *PIK3CA*, *PTEN*, *PTPN11*, *RB1*, *RET*, *SMAD4*, *SMARCB1*, *SMO*, *SRC*, *STK11*, *TP53*, and *VHL*. Then, when we initiated the sequencing of ccfDNA extracted from blood, we had to switch to another cancer panel, as the Illumina panel was not appropriate for short DNA sequences. We therefore selected the 56G Oncology Panel V2 from Swift Biosciences (Ann Arbor, MI) that contained the same gene targets covered by the Illumina kit, as well as 8 additional genes absent in the Illumina cancer panel (and therefore not included in our comparative analysis). Per sample, we only considered a mean coverage of at least 1000× for genomic DNA and of at least 500× for blood DNA sequencing. For the ccfDNA data, we only considered SNV and indel variants at 1% allelic frequency or higher in target regions with sufficient read coverage (at least 100×).

### 2.4. Data Analysis

Sequencing data derived from the Illumina TruSeq Amplicon Cancer Panel was analyzed using the Illumina BaseSpace platform (TruSeq Amplicon analysis). ccfDNA data obtained using the 56G Oncology Panel V2 was analyzed using Genialis Expressions (Accel-Amplicon analysis workflow, Genialis Inc., Boston, MA, USA). In brief, quality trimmed (Trimmomatic v.0.36) sequencing data were aligned to the human genome (GRCh37 assembly) using BWA MEM (v. 0.7.17-r1188). The aligned data were further processed by trimming primer sequences (Primerclip, Swift biosciences) and GATK (v.3.6) tools (IndelRealigner and BaseRecalibrator) to prepare analysis ready BAM file. SNP/INDELs were called using LoFreq (v.2.1.3.1) and annotated using snpEff (v.4.3k).

## 3. Results

### 3.1. Selection of Genomic DNA/Plasma DNA Pairs with Interpretable Data

We aimed to determine if NGS could identify similar mutations in tumor DNA and plasma DNA obtained from nine cancer types. We extracted the DNA from tumor tissues obtained from 209 patients. After quality control, the samples were sequenced (using the 48-gene panel from Illumina and our in-house MiSeq sequencer) to identify cancer-driving gene mutations. Then, as a second step in these patients, we also extracted DNA from their plasma samples. Blood samples were collected at time of tumor collection for the following cancer types: breast *n* = 10, cholangiocarcinoma *n* = 5, colorectal *n* = 24, glioblastoma *n* = 38, liver *n* = 12, lung *n* = 18, melanoma *n* = 48, ovarian *n* = 31, and pancreas *n* = 23. Since the Illumina panel was not appropriate for short DNA fragments, extracted ccfDNAs were sequenced using the 56-gene panel from Swift and our in-house MiSeq sequencer.

We validated the Swift panel by sequencing some genomic DNA samples previously extracted and sequenced using the Illumina panel, and we found no difference in the pathogenic mutations detected or in the variant allele frequency (VAF) for individual mutations. For example, the *BRAF* V600E mutation in MM-348 found at 73% VAF using the Illumina cancer panel (Table S1) was detected at 74% VAF using the Swift cancer panel, the *EGFR* S715I mutation in LNGCA-0002 was found at 5% in both panels, and, in the CRC-0015, the three pathogenic mutations, *APC* Y1376fs, *KRAS* G12D, *TP53* T211I, were found at 32%, 63%, 56% (respectively) using the Illumina panel, and 32%, 68%, 55% using the Swift panel.

After sequencing, all the FASTQ files were downloaded from the MiSeq machine, analyzed by Illumina for the tumor DNA samples, or transferred to a company website for the ctDNA samples. This issue regarding the secure data storage and reproducible data analysis has to be considered when the experiments are performed in a small institution that lacks appropriate bioinformatics support. After careful consideration, Illumina BaseSpace and Genialis Expressions software were used to analyze data from tumor DNA and plasma DNA samples, respectively.

It is important to note that data from almost half of the pairs, corresponding to a total of 102 patients, were uninformative. There was either no driver mutation detected in the original tumor (about 2/3 of these 102 samples) or technical issues were encountered during sequencing, incorrect tumor type, or lack of information about cancer stage (about one-third of the 102 samples with uninterpretable data). The pairs with interpretable data were related to 107 patients and were as follows: breast (BRC) *n* = 4, cholangiocarcinoma (CHNG) *n* = 2, colorectal (CRC) *n* = 14, glioblastoma (GBM) *n* = 19, liver (LVRCA) *n* = 4, lung (LNGCA) *n* = 12, melanoma (MM) *n* = 27, ovarian (OVCA) *n* = 15, and pancreas (PANC) *n* = 10.

Therefore, and as described in the Discussion section, to maximize the number of matched pairs that can be analyzed, before embarking on a blood collection, it may be advisable to first determine the presence of driver mutation(s) in the tumor sample that can be detected using the cancer panel of choice. For example, most of the commercially available panels, such as the ones we used, do not allow the identification of gene amplifications or large deletions.

### 3.2. Some Tumor Types Are More Appropriate Than Others for ctDNA Analysis

**Figure 1:**
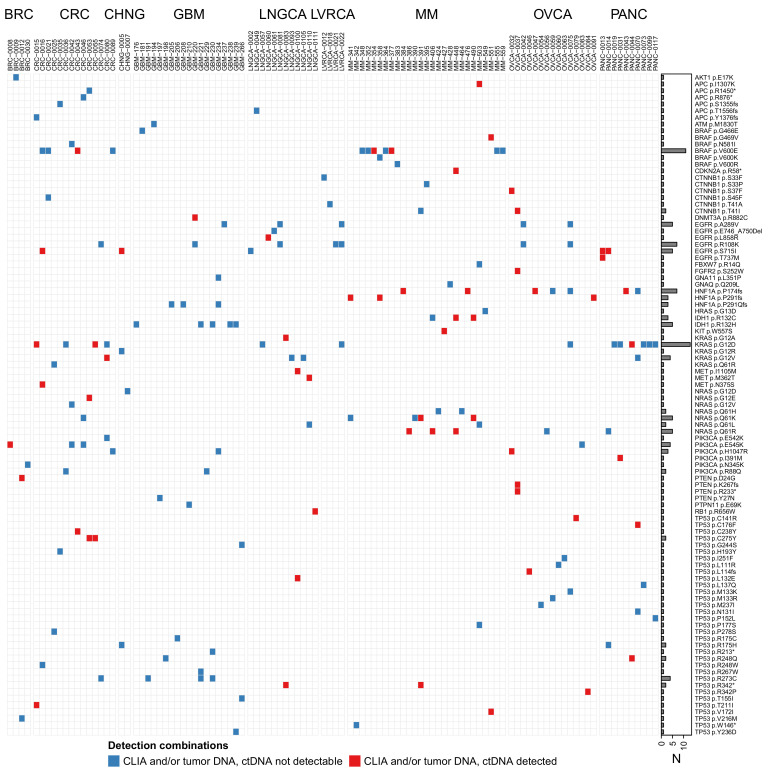
A total of 107 human samples investigated are listed in the *X*-axis, with 99 mutations detected listed in the *Y*-axis. The pairs with circulating tumor DNA (ctDNA) not detectable in the blood are indicated in blue, and the pairs with ctDNA detectable in the blood are indicated in red. Tumor mutations were identified by a CLIA test (such as Foundation One) and/or by our in-house MiSeq sequencer.

**Figure 2:**
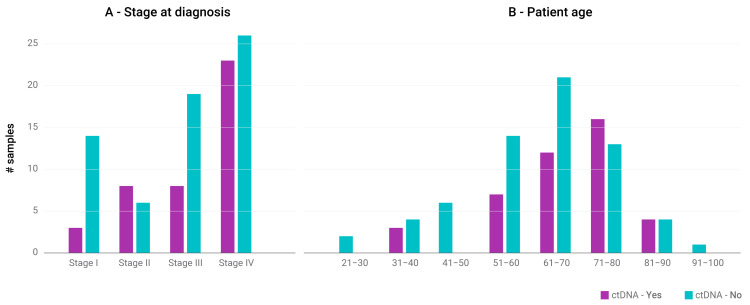
Correlation between sample count (with or without ctDNA presence) and (**A**) stage at diagnosis, (**B**) patient age.

Analysis of 48 gene sequences common to both panels was performed and revealed that about 40% of the 107 sample pairs contained at least one pathogenic/driver mutation detected in the DNA extracted from plasma (Figure 1, Table 1, and Table S1). The ratio was close to 50% when the GBM samples were excluded. By considering the individual cancer types, and therefore a limited number of samples, we determined that melanoma, ovarian cancer, lung cancer, colorectal cancer, and pancreatic cancer were most likely to shed ctDNA to the bloodstream, particularly in patients with stage IV disease (Figure 2A). Intriguingly, two out of three stage I patients with detectable ctDNA had pancreatic cancer. The ratio of pathogenic mutations in the blood was also higher in older patients (specifically age 71–80) (Figure 2B). Even though the majority of the GBM patients had a primary tumor with pathogenic mutations at high VAF (above 30%), except for one case, we could not detect ctDNA in plasma samples, which is in agreement with the literature [12]. This may be due to the presence of the blood–brain barrier. For the rest of the tumor types, i.e., liver cancer, cholangiocarcinoma, and breast cancer, we did not analyze enough samples to reach firm conclusions regarding the potential yield of liquid biopsies for patients at a particular age or disease stage.

While setting up an NGS facility in the context of a medical center, it may be wise to focus on specific tumor types rather than extracting blood ccfDNA from multiple cancer types such as GBM (where ccfDNA are as abundant as for other types of cancer, but may only correspond to fragments of DNA shed by normal/blood cells), where pathogenic mutations in ctDNA are better detected in the cerebrospinal fluid [12]. Moreover, selecting samples from too many cancer types can hinder the use of statistics to draw conclusions for any given cancer type, and this is the reason why we recommend the selection of limited tumor types.

### 3.3. Case Reports Associated with Our Liquid biopsy Study

An ultimate goal of this study is to select cancer patients with specific types of tumors who would benefit from longitudinal studies of their blood for disease monitoring and treatment response. As examples, we list here some case reports, which could be present in studies besides ours, to support the promise of sequencing DNA extracted from plasma samples.

Besides the direct comparison between tumors and blood samples harvested at time of surgery, liquid biopsies can be used in longitudinal studies in a non-invasive manner, monitoring an individual patient’s response to treatment. We recently initiated this line of investigation in our institute and we report here a few preliminary cases. For example, patient PANC-0117 had stage I pancreatic cancer at the time of surgery, with two pathogenic mutations present in the tumor (*KRAS* G12D and *TP53* P152L) that were not detectable in the plasma sample (Table 2). These two mutations were confirmed to drive the tumor since they were also present in DNA extracted from primary patient-derived xenografts (PDXs) (PANC-0117-X and then PANC-0117-X2). Another blood sample obtained three months later from the same patient (PANC-0130) was also negative for both mutations; however, after one and a half years, when the patient progressed to a more advanced stage, the plasma sample was then positive for both original pathogenic mutations (PANC-0146) (Table 2).

A second case report that we are highlighting is LNGCA-003, which was not included in the pair-wise comparisons because the VAF in the tumor was less than 5%. However, the *EGFR* mutation S715I present in the tumor at 1.3% was detected in ctDNA at 4.4%. It is therefore important to consider the threshold of the VAF mutation present in the DNA extracted from tissue since tumors can be highly heterogeneous and clonal expansion of particularly aggressive cancer cell subpopulations, whose mutations are then detectable in the plasma, could be responsible for the worsening of the disease.

As a third case report, we present patient CRC-0042 (stage II) who had the following mutations detectable by sequencing of the original tumor: *BRAF* (N581I at 28.7% VAF), *NRAS* (G12V at 41.9%), and *PIK3CA* (E545K at 57.7%). Even though there were several driver mutations in the tumor tissue, none of these mutations was detected in the blood (Figure 1 and Table S1). In two subsequent longitudinal blood samples (CRC-0071 and CRC-0098), neither of these mutations was detectable and no new driver mutation was identified. Even though there was progression to stage IV disease, the patient is still alive three years after surgery.

As a fourth case report, patient CRC-0043 (stage IV) had the following mutations found by sequencing the original tumor: *BRAF* (V600E at 35.5% VAF) and *TP53* (C238Y at 56.2%). Both mutations were detected in the blood collected at the time of surgery, *BRAF* (V600E at 7.1%) and *TP53* (C238Y at 12.5%) (Figure 1 and Table S1). However, in a subsequent blood sample (CRC-0067) collected four months after surgery and during FOLFOX (folinic acid, fluorouracil, and oxaliplatin) treatment, none of these pathogenic mutations was detected and no new driver mutation was present, indicating that the patient probably initially responded to the treatment (Table 3). Interestingly, in a subsequent blood sample (CRC-0094) collected six months after the second collection, at a time the patient had to be switched to bevacizumab treatment, while the two original mutations were still undetectable, a new driver mutation was detected in the *KRAS* gene (G60D at 1.2%). This patient with a stage IV colorectal cancer was deceased soon after this last blood collection in which the *KRAS* G60D mutation was identified.

## 4. Discussion

Successful mastery of the techniques needed for liquid biopsy studies may soon enable the monitoring of patients using serial blood samples, without the need for additional biopsies or surgery [1]. ctDNA-based approaches targeting various commonly mutated genes has shown promise in detecting tumor burden in metastatic cancer patients. However, the analysis of pathogenic/driver mutations in the ctDNA extracted from blood samples requires several steps that should be carefully considered when setting up a sequencing laboratory at a single institution. Before embarking on a blood collection, it may be advisable to first determine the presence of driver mutation(s) in the tumor sample that can be detected using the cancer panel of choice, with pathogenic mutations being prioritized over benign mutations. As ctDNA represents a small fraction of the total blood ccfDNA, sensitive detection methods are necessary. Next-generation sequencing is a promising tool for rapidly characterizing multiple mutations across a large number of genes in both tumor and plasma DNA. Even though there are CLIA-certified and FDA-approved assays such as Guardant 360 (guardant360cdx.com, accessed on 10 June 2022), there are no standardized assays to comprehensively detect pathogenic/driver mutations in ctDNA, therefore justifying the rationale for additional research.

We used commercially-available NGS cancer gene panels for laboratory implementation, but there are also alternative approaches to detect ctDNA mutations, with the differences between the various liquid biopsy platforms being mainly related to levels of mutation detection. Chang et al. determined the levels of mutant *BRAF* and *NRAS* ctDNA in melanoma patients using droplet digital PCR (ddPCR) [13]. Overall, ctDNA had a higher sensitivity than lactate dehydrogenase (LDH) to detect disease progression. The same group recently demonstrated the potential of probe-based ddPCR assays to specifically detect and quantify *TERT* promoter mutations in tumors and plasma of melanoma patients [14]. The limits of detection for two *TERT* mutations (at positions −124 and −146) were 0.062% and 0.051% mutant allele fraction. Another group investigated the prognostic impact of ctDNA in melanoma patients [15]. ddPCR assays were performed to detect *BRAF/NRAS* mutations in plasma from 161 stage II/III melanoma patients enrolled in the AVAST-M adjuvant trial. Patients with detectable ctDNA had significantly decreased disease-free and overall survival, which was validated in independent cohorts [16].

Using either Illumina or Swift kits for target amplification and library construction, sequencing was performed using our in-house Illumina MiSeq platform. We found that some key pathogenic mutations identified in the solid tumor were also present in ctDNA. The overall concordance between tumor DNA and plasma ctDNA mutation frequencies varied widely by tumor type. The greatest concordance was observed for pancreatic cancer (6/10, 60%), followed by melanoma (14/27, 52%), cholangiocarcinoma (1/2, 50%), breast cancer (2/4, 50%), ovarian cancer (7/15, 46%), colorectal cancer (6/14, 43%), lung cancer (5/12, 42%), glioblastoma (1/19, 5%), and finally liver cancer (0/4, 0%). In the tumor types amenable to this type of analysis, mutational profiling of longitudinal blood biopsies will enable us to monitor each cancer patient’s response to treatment and/or evolution toward drug resistance in real-time and in a non-invasive manner. This information will be instrumental in designing treatment strategies in patients with recurrent or progressive disease.

ctDNA in the peripheral blood is a liquid biopsy that contains representative tumor information, including gene mutations representative of those found in primary tumors [1]. The specific genetic changes found in ctDNA can have diagnostic value and predict response to treatment and patient survival. Additionally, as liquid biopsies are easily obtainable, repeated samples can be collected for real-time monitoring of both cancer patients’ response to treatment and to detect disease progression over time. As such, peripheral blood liquid biopsies that contain tumor-representative ctDNA have been proposed as an alternative to solid tumor biopsies.

In conclusion, the present study shows that the identity of the ctDNA pathogenic mutations found in the blood correlates with those found in primary tumor DNA for most cancer types. These results need to be further validated in larger studies and matched with clinical outcome data in order to fully demonstrate the utility of this targeted sequencing method in a diagnostic setting. Moreover, somatic mutations are also commonly found in healthy individuals, which interfere with the effectiveness of cancer diagnostics. Investigating 821 samples, a study showed that most of the mutations in ccfDNA were highly correlated to mutations present in the DNA of white blood cells [17]. This specific study reported frequencies significantly lower than us (our threshold was 1%), and caution needs to be taken for blood-prone mutations when using ccfDNA as a diagnostic tool. Therefore, including the extraction and sequencing of the DNA from buffy coat after centrifugation of the blood is advisable. Overall, our study describes the advantages and hurdles of setting up a liquid biopsy ctDNA approach without the use of more expensive CLIA-approved technologies, which are still unavailable in many countries and centers.

## Figures and Tables

**Table 1:** Aggregated per-gene mutation events across different tumor types showing number of mutation events detected in both tumor DNA and plasma DNA samples versus total count of detected mutations in tumor DNA samples.

Gene	BRC	CHNG	CRC	GBM	LNGCA	LVRCA	MM	OVCA	PANC
TP53	0/1	0/1	4/8	0/7	2/2	/	2/4	3/8	2/5
HNF1A	/	/	/	0/3	/	/	4/4	2/4	1/2
NRAS	/	0/1	1/3	/	0/1	/	5/10	0/1	0/1
EGFR	/	1/1	1/2	0/2	1/4	0/2	/	0/2	2/2
KRAS	/	0/1	3/5	/	1/4	0/1	/	0/1	1/7
BRAF	/	/	1/5	0/1	/	/	3/10	/	/
MET	/	/	1/1	/	2/2	/	/	/	/
PIK3CA	1/2	/	0/5	0/2	/	/	/	1/2	1/1
CTNNB1	/	/	0/1	/	/	0/2	0/2	2/2	/
IDH1	/	/	/	0/5	/	/	2/3	/	/
PTEN	1/1	/	/	0/1	/	/	/	1/1	/
APC	/	/	0/4	/	0/1	/	1/1	/	/
CDKN2A	/	/	/	/	/	/	1/1	/	/
DNMT3A	/	/	/	1/1	/	/	/	/	/
FGFR2	/	/	/	/	/	/	/	1/1	/
KIT	/	/	/	/	/	/	1/1	/	/
RB1	/	/	/	/	1/1	/	/	/	/
AKT1	0/1	/	/	/	/	/	/	/	/
ATM	/	/	/	0/1	/	/	/	/	/
FBXW7	/	/	/	/	/	/	0/1	/	/
GNA11	/	/	/	0/1	/	/	/	/	/
GNAQ	/	/	/	/	/	/	0/1	/	/
HRAS	/	/	/	/	/	/	0/1	/	/
PTPN11	/	/	/	0/1	/	/	/	/	/
Matched Σ:	2/5	1/4	11/34	1/25	7/15	0/5	19/39	10/22	7/18

**Table 2:** Case report corresponding to a pancreatic cancer patient from whom three longitudinal blood samples were collected and analyzed for the presence of pathological mutations (ND = not detectable).

Samples Related to Patient PANC-0117	Date of Collection	Mutation(s) Detected	Variant Allele Frequency	Stage of the Disease and Status
PANC-0117 original tumor	27 April 2018	KRAS (G12D) TP53 (P152L)	17.1% 24.9%	Stage IB and stable disease
PANC-0117-X tumor		KRAS (G12D) TP53 (P152L)	39.1% 66.4%	
PANC-0117-X2 tumor		KRAS (G12D) TP53 (P152L)	46.3% 88.5%	
PANC-0117-ctDNA	27 April 2018	ND ND		
PANC-0130-ctDNA	12 July 2018	ND ND		
PANC-0146-ctDNA	8 January 2020	KRAS (G12D) TP53 (P152L)	12% 9.7%	Stage IV and disease progression

**Table 3:** Case report corresponding to a colorectal cancer patient from whom three longitudinal blood samples were collected and analyzed for the presence of pathological mutations (ND = not detectable).

Samples Related to Patient CRC-0043	Date of Collection	Mutation(s) Detected	Variant Allele Frequency	Stage of the Disease and Status
CRC-0043 original tumor	3 March 2017	BRAF (V600E) TP53 (C238Y)	35.5% 56.2%	Stage IV
CRC-0043-ctDNA	3 March 2017	BRAF (V600E) TP53 (C238Y)	7.1% 12.5%	
CRC-0067-ctDNA	18 July 2017	ND		Stable disease
CRC-0094-ctDNA	11 January 2018	KRAS (G60D)	1.2%	Disease progressed and patient deceased

## Data Availability

The authors confirm that the data supporting the findings of this study are available within the article.

## Supplementary Materials

The following supporting information Table S1: Supplementary Table S1 can be downloaded at: https://www.mdpi.com/article/10.3390/jpm12111845/s1.

## Abbreviations

ccfDNA, circulating cell-free DNA; ctDNA, circulating tumor DNA; CRC, colorectal cancer; GBM, glioblastoma; indel, insertion/deletion; NGS, next-generation sequencing; SNV, single-nucleotide variant; VAF, variant allele frequency.
